# Changes in pain scores and walking distance after epidural steroid injection in patients with lumbar central spinal stenosis

**DOI:** 10.1097/MD.0000000000029302

**Published:** 2022-06-17

**Authors:** Minsoo Kim, Soyeon Cho, Yeonji Noh, Daehun Goh, Hee-Jeong Son, Jin Huh, Seong-Sik Kang, Byeongmun Hwang

**Affiliations:** Department of Anesthesiology and Pain Medicine, Kangwon National University Hospital, School of Medicine, Kangwon National University, Republic of Korea.

**Keywords:** epidural steroid injection, low back pain, spinal stenosis, walking

## Abstract

Lumbar spinal stenosis is a common degenerative disorder that is characterized by pain and neurogenic claudication. Previous studies have evaluated the effects of an epidural steroid injection (ESI) on spinal stenosis, based on changes to the spinal canal diameter.

This study aimed to examine the impact of the ESI on pain scores and walking distance in patients with lumbar central spinal stenosis, stratified based on disease severity, which was graded according to the degree of cauda equina separation.

We reviewed the medical records of patients who received the ESI for lumbar spinal central canal stenosis. A total of 128 patients were divided into moderate and severe groups, based on the degree of cauda equina separation.

Relative to baseline values, 2 weeks after the ESI, the moderate group showed a significant decrease in the numeric rating scale (NRS) scores and an increase in walking distance. Meanwhile, the severe group showed a significant decrease in the NRS scores and no significant change in walking distance. The moderate group had lower NRS scores and a longer walking distance than did the severe group 2 weeks after the ESI. The proportion of patients with improved levels of satisfaction was higher in the moderate group than in the severe group.

Lumbar interlaminar ESI may reduce pain scores and increase walking distance in patients with moderate lumbar spinal central canal stenosis. Patients with moderate spinal stenosis achieved better outcomes than did patients with severe stenosis.

## 1 Introduction

Lumbar spinal stenosis (SS) is a common degenerative disorder that is characterized by pain and neurogenic claudication.^[1]^ Treatment for SS includes patient education, medication, physical therapy, and exercise.^[2]^ An epidural steroid injection (ESI) may be considered in patients whose condition does not improve with conservative care.^[3]^ While some studies have challenged the role of the ESI in pain reduction, functional improvement, and claudication in patients with lumbar SS,^[4–6]^ other studies have suggested that the ESI may help relieve pain and improve functional outcomes in these patients.^[7–9]^ Consequently, the effects of the ESI on lumbar SS remain subject to debate,^[9–11]^ while the effectiveness of treatment for lumbar SS may vary depending on disease severity.

Lumbar SS refers to the symptoms associated with the anatomical reduction of the lumbar spinal canal size. However, some individuals have a markedly narrowed canal without developing any symptoms, which raises the question of the role of central canal stenosis in symptom development. Moreover, the associations among dural sac stenosis and the numeric rating scale (NRS) pain scores or functional capacity (e.g., walking distance) are not straightforward.^[12,13]^ Previous studies have reported that the spinal canal dimension does not predict the success or failure of the ESI in patients with lumbar SS, and that the spinal canal size is not significantly associated with clinical symptoms among these patients.^[10,12]^ Furthermore, previous studies have reported that the effects of the ESI are not associated with SS severity, based on spinal canal diameter assessment.^[14,15]^

Recent studies have proposed magnetic resonance imaging (MRI)-based assessment of the degree of cauda equina separation as an alternative method for disease severity measurement.^[16]^ The impact of the ESI on SS assessed using the novel criteria may be different from that assessed using conventional measures. In fact, some studies based on this new grading system have shown that the ESI may be an effective pain management tool in lumbar SS.^[17,18]^ However, these studies assessed only the levels of pain associated with the degree of cauda equina separation rather than walking ability.

Neurogenic claudication due to lumbar SS may impair walking ability in older adults.^[1]^ As walking is critical to overall health, impaired walking ability may increase the risk of cardiovascular disease, thromboembolic stroke, diabetes mellitus, and cognitive disorders in older adults;^[19–21]^ thus, restoring walking ability should be a treatment goal alongside pain management in patients with lumbar SS. In addition, adequate assessment of ambulation-related disability and levels of pain in patients who receive ESI is paramount to assessing treatment effectiveness. To the best of our knowledge, no previous study has investigated the effects of the ESI on pain scores and walking distance in patients stratified according to SS severity, assessed using the novel grading system. This study aimed to assess the impact of the ESI on pain scores and walking distance in patients with lumbar central stenosis, stratified according to the degree of cauda equina separation.

## 2 Methods

### 2.1 Study population

This study involved a retrospective analysis of data from patients who received an ESI for lumbar SS at the pain management practice center of the Kangwon National University Hospital between July 2015 and November 2020. The study was approved by the institutional review board (No. KNUH-2020–12-016-002) and was conducted in accordance with the Declaration of Helsinki.

This study included adults aged ≥50 years who received the ESI for management of lumbar spinal stenosis, experienced walking impairment due to lumbar SS, and had moderate or severe central canal stenosis in the lumbar spine MRI scan within 6 months before the ESI. The exclusion criteria were a presence of severe foraminal stenosis or herniated intervertebral disc with radiculopathy in the lumbar region; previous surgery to the lumbar spine or femur; administration of spinal corticosteroid injection within 6 months; or presence of any other disorders that could limit ambulation, including severe osteoarthritis of the hip or knee, severe cardio-vascular disease, chronic obstructive pulmonary disease, and neurologic deficits. Overall, 128 patients were included in this study and divided into moderate and severe SS groups after age-based (± 2 years) matching, including 64 patients per group. The following information was collected based on reviews of the participants’ medical records: medical history, pain scores, patient satisfaction scores, and intermittent claudication distance.

**Figure 1:**
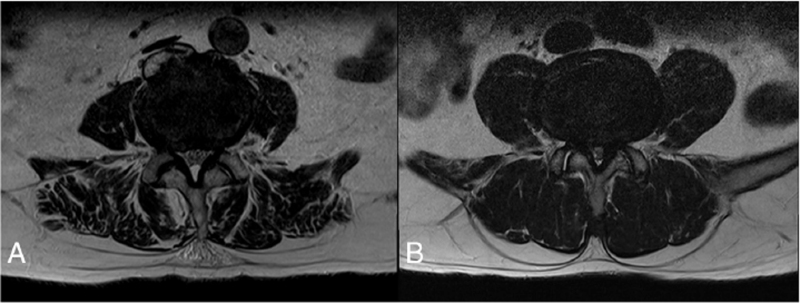
Grading system for lumbar central spinal stenosis based on T_2_-weighted axial magnetic resonance imaging findings of the lumbar spine. Lumbar central canal stenosis is present when the anterior cerebrospinal fluid space is obliterated and divided; moderate stenosis refers to the aggregation of some cauda equina, (a); severe stenosis refers to a state whereby the entire cauda equina appears as a bundle (b).

Lumbar SS was diagnosed based on medical history, including clinical symptoms, and physical, neurologic, and MRI examination findings. The SS diagnostic criteria were based on the North American Spine Society guidelines.^[22]^ The severity of the spinal central canal stenosis was assessed by a radiologist blinded to other study data, and classified as moderate and severe. The “moderate” stenosis category referred to the aggregation of some cauda equina, and “severe” stenosis referred to the absence of cauda equina separation, which created a bundle-like appearance (Fig. 1).^[16]^ MRI scanning was performed with a 1.5 Tesla scanner, using a spine surface coil, and included T_1_-weighted axial imaging sequences and T_2_-weighted sagittal imaging sequences. The patients also received medications, including nonsteroidal anti-inflammatory drugs, muscle relaxants, neuromodulators (pregabalin, and/or gabapentin), and limaprost.

The patients’ pain levels were assessed using the NRS, with scores ranging from 0 (no pain) to 10 (worst pain) points.^[15,23]^ A decrease in the NRS score of ≥2 points was considered indicative of a therapeutic effect.^[24]^ The patients’ walking capacity was expressed as a walking distance, ^[1,25,26]^ which was measured as the maximum distance that a person could walk on a walking track at their own pace, without taking a break. Patients walked continuously until they were forced to stop or felt the need to stop due to the symptoms of SS. The NRS scores and walking distance were measured at baseline (before the ESI) and at 2 weeks after the ESI, to assess treatment effects. The degree of patient satisfaction was assessed using the Roland 5-point patient satisfaction scale (0, 1, 2, 3, 4, and 5 points, representing absence of pain, little pain, moderate pain, bad pain, very bad pain, and nearly unbearable pain, respectively) at 2 weeks after the ESI.^[18]^ Patients with Roland scale score of 0–2 points were considered “successful responders with improvement”.

### 2.2 Epidural steroid injection

In this study, we used an interlaminar ESI in patients with lumbar central stenosis. Weil et al. ^[27]^ have suggested that a contrast agent may be distributed to more than one vertebral level, independent of the needle location, suggesting that an interlaminar ESI may be more beneficial than a transforaminal ESI in patients with central SS, as a higher volume of the injectate can be administered and distributed to a broader area with the former than with the latter method. Consequently, we used interlaminar ESI in our study, as did Sencan et al..^[15]^ The ESI was performed as previously described.^[24]^ Briefly, all patients received lumbar ESI at the most stenotic level under fluoroscopic imaging, using the interlaminar method. The ESI included 8 mL lidocaine hydrochloride (0.5%, preservative-free) and 5 mg dexamethasone, and was administered at the lumbar spine level. Two weeks thereafter, all patients returned to the clinic for treatment outcome assessments. No other interventions were offered to the study patients. However, the patients continued therapy with non-opioid analgesics and pursued normal activities, including work.

### 2.3 Statistical analysis

Statistical analyses were performed using SPSS 25.0 (IBM Corporation, Armonk, NY). The data were presented as mean ± standard deviation or count (%). Among-group comparisons of demographic and clinical characteristics were performed using the Student t-test. Within-group comparisons of the parameters of interest at baseline and follow-up were performed using the paired t-test. The chi-square test was used to compare the differences in categorical variables among the groups. The normal distribution assumption of the data was verified with the Levene test. In all the comparisons, *P*-values of < 0.05 were considered statistically significant.

## 3 Results

A total of 128 patients were included in this study and divided into moderate and severe SS groups, including 64 patients per group. The patients’ baseline characteristics are presented in Table 1. There was no significant difference in the demographic or clinical characteristics among the groups (Table 1).

**Table 1:** Clinical and demographic characteristics of the study population.

Characteristic	Severity of spinal stenosis		
	Moderate group (n = 64)	Severe group (n = 64)	*P* values
Age (y)	69.2 ± 9.3	69.6 ± 8.2	0.803
Sex (male, %)	36 (56%)	31 (51%)	0.376
BMI (kg/m^2^)	24.3 ± 2.8	23.7 ± 2.6	0.514
Duration of symptoms (y)	6.4 ± 4.2	6.2 ± 5.2	0.417
Medications			
NSAIDs	43 (67%)	42 (66%)	0.852
Muscle relaxants	29 (45%)	28 (44%)	0.859
Neuromodulators	8 (13%)	10 (16%)	0.611
Limaprost	54 (84%)	58 (91%)	0.285
Injection sites			
L2/3	2 (3%)	2 (3%)	1.000
L3/4	20 (31%)	18 (28%)	0.699
L4/5	40 (63%)	43 (67%)	0.579
L5/S1	2 (3%)	1 (2%)	0.559

Values are presented as mean ± standard deviation or count (%). The “moderate” and “severe” groups included patients with moderate and severe lumbar spinal stenosis, respectively, who received an epidural steroid injection. BMI, body mass index; NSAID, Non-steroid anti-inflammatory drugs. Differences were considered statistically significant at *P*-values of < 0.05.

The proportion of patients with improvement in the degree of satisfaction was higher in the moderate group than in the severe group (*P* < 0.001, Table 2). At 2 weeks after the ESI, the proportion of patients with an improvement in the degree of satisfaction in the moderate and severe groups was 64% and 31%, respectively (Table 2).

**Table 2:** Patient satisfaction ratings.

Variable	Moderate group (n = 64)	Severe group (n = 64)
0–1	15 (23%)	4 (6%)
2	26 (41%)	16 (25%)
3	13 (20%)	30 (47%)
4–5	10 (16%)	14 (22%)
Improvement	41 (64%)^∗^	20 (31%)

Values represent counts (%). The “moderate” and “severe” groups included patients with moderate and severe lumbar spinal stenosis, respectively, who received an epidural steroid injection. Patients with Roland scale 0–2 in the rating were considered successful responders with improvement.

∗ P-value of < 0.05 compared with the severe group.

The baseline mean NRS scores in the moderate and severe groups were 7.1 ± 1.2 and 7.2 ± 1.1 points, respectively, without any significant differences. However, 2 weeks after the ESI, the mean NRS scores in the moderate and severe groups decreased to 4.3 ± 1.8 and 5.8 ± 1.7 points, respectively, compared with baseline values, marking a ≥2-point decrease in the moderate group (*P* < 0.001, Table 3). In addition, 2 weeks after the ESI, NRS pain scores were lower in the moderate group than in the severe group (*P* < 0.001, Table 3).

**Table 3:** Changes in pain intensity, evaluated using numeric rating scale (NRS) scores.

	Numeric rating scale score (points)		
	Moderate group	Severe group	*P-*value^a^
Baseline	7.1 ± 1.2	7.2 ± 1.1	0.414
2 weeks after ESI	4.3 ± 1.8	5.8 ± 1.7	< 0.001
*P-*value^b^	< 0.001	< 0.001	
Change of intragroup mean value	2.8	1.4	

The “moderate” and “severe” groups included patients with moderate and severe lumbar spinal stenosis, respectively, who received an epidural steroid injection. Values are presented as mean ± standard deviation.

a Student t-test (for comparison of mean values between groups; *P*-values of < 0.05 were considered significant).

b Paired t-test (for comparison of intragroup mean values, *P*-values of < 0.05 were considered significant).

The mean baseline walking distance values in the moderate and severe groups were 341 ± 272 and 328 ± 266 meters, respectively, without any significant differences. However, 2 weeks after the ESI, the mean walking distance values in the moderate and severe groups were 448 ± 296 and 345 ± 284 meters, respectively. Compared to baseline values, 2 weeks after treatment, the moderate group showed a significant increase in walking distance (*P* < 0.001, Table 4); meanwhile, the severe group showed no change in walking distance. Finally, 2 weeks after the ESI, the moderate group had a longer walking distance than the severe group (*P* < 0.001, Table 4).

**Table 4:** Changes in walking distance.

	Walking distance, meters		
	Moderate group	Severe group	*P-*value^a^
Baseline	341 ± 272	328 ± 266	0.523
2 weeks after ESI	448 ± 296	345 ± 2284	< 0.001
*P-*value^b^	< 0.001	0.094	

The “moderate” and “severe” groups included patients with moderate and severe lumbar spinal stenosis, respectively, who received an epidural steroid injection. Values are presented as mean ± standard deviation.

a Student t-test (for comparison of mean values between groups; *P*-values of < 0.05 were considered significant).

b Paired t-test (for comparison of intragroup mean values, *P*-values of < 0.05 were considered significant).

## 4 Discussion

In this study, we examined the impact of the ESI on pain scores, walking distance, and satisfaction levels of patients with lumbar SS, graded according to the degree of cauda equina separation. Two weeks after the ESI, the moderate group showed a significant decrease in the NRS scores and an increase in walking distance, while the severe group showed no change to walking distance, compared to the corresponding baseline values. Moreover, 2 weeks after treatment, the moderate group had lower NRS scores and longer walking distance than did the severe group. Furthermore, the degree of satisfaction was higher in the moderate group than in the severe group.

Previous studies have reported that the severity of SS is independent of the effects of ESI, based on spinal canal diameter assessment.^[10,12,13]^ Cosgrove et al. have shown that the ESI may be an effective treatment for improving ambulation and functional limitations associated with lumbar SS; however, MRI-based disease severity findings have not been associated with a favorable response to the ESI.^[28]^ It should be noted that Cosgrove et al. used the older SS classification method,^[28]^ based on the anteroposterior diameter of the spinal canal. Meanwhile, the results of our study suggest that the grading of disease severity based on the degree of cauda equina separation is associated with a favorable response to ESI, and may be useful in predicting the effects of ESI in patients with lumbar central stenosis.

Few studies have assessed the severity of lumbar central stenosis based on the degree of cauda equina separation.^[17,18]^ Although it is difficult to make a direct comparison with Do et al.'s findings, our study has also shown improved NRS pain scores in the moderate group.^[17]^ In the study by Do et al., the NRS pain scores decreased after the ESI, and 30% and 18% of the patients with moderate and severe stenosis experienced pain relief (pain score reduction of ≥50%), respectively.^[17]^ In our study, the NRS pain scores decreased after the ESI; however, a decrease of the NRS score in the moderate and severe groups was 2.8 and 1.4 points, respectively (≥2 points considered indicative of a therapeutic effect).^[24]^ Moreover, the proportion of patients that experienced pain relief after the ESI was 64% and 31% in group with moderate and severe stenosis, respectively. The higher proportion of patients that experienced pain relief after the ESI in the present than in the previous study by Do et al. might have been due to the higher initial pain score among the patients in this study; the corresponding baseline NRS scores were 7.1 and 4.6 points, respectively.^[17]^ The patients in our study might have been more satisfied with the effects of the ESI due to higher baseline pain levels. Furthermore, in the study by Do et al., the reduction in pain scores was more pronounced in the patients with moderate SS than in those with severe SS; this finding was consistent with that of our study. Moreover, the group with severe SS in our study showed poorer outcomes than that with moderate SS. This finding may be accounted for by the SS classification criteria used, which consider the extent of nerve compression in symptomatic patients. Meanwhile, in a study by Park et al., the ESI provided effective pain relief in lumbar SS; however, the grade of lumbar SS appeared unrelated to the degree of pain relief obtained with the ESI.^[18]^ This discrepancy in findings might have been due to the small number of patients with severe SS who were included in the study by Park et al. (n = 6).

Walking difficulty is a characteristic of lumbar SS in patients with neurogenic claudication, where improved walking ability is the primary treatment goal. However, treatments to improve walking capacity remain controversial.^[4,5,26]^ Recently, Sencan et al. have reported that walking distance increases in patients with lumbar central stenosis following the administration of an interlaminar ESI.^[15]^ Our findings were consistent with those of this study, which might have been due to the decrease in peri-neural inflammation, edema, and pain levels. The present study is first to investigate whether SS severity graded according to the degree of cauda equina separation affects walking ability after the ESI in patients with lumbar central stenosis. In the present study, walking distance increased in the moderate group after the ESI; however, it remained unchanged in the severe group. This finding suggests an association between walking ability following the ESI and the grading of SS severity. The lack of change in this parameter in the severe group suggests that severe SS may be an advanced disease; this observation is further supported by the shorter walking distance observed in this group than that observed in the moderate group.

In previous studies, the proportion of patients with lumbar SS reporting improvements after the ESI was in the range of 52–77%.^[8,25]^ In this study, the corresponding value was 48%. The relatively low proportion of patients showing improvement in this study may be due to a higher proportion (50%) of patients with severe stenosis in this than in the previous studies. The proportion of patients with improvement in the moderate and severe groups was 64% and 31%, respectively, with the lowest number of patients reporting improvement in the severe group. Based on these findings, pain management physicians should inform patients with severe SS that the effectiveness of an ESI in their condition may be unsatisfactory.

The impact of the ESI on lumbar SS remains controversial.^[6,7,10,11]^ However, clinical trial and systematic review findings suggest that the ESI may help relieve pain and improve functional outcomes in patients with lumbar SS.^[6–9]^ Previous studies have reported an NRS score decrease of 3.1–6 points within 6 weeks to 3 months after an interlaminar ESI in patients with lumbar central stenosis.^[6–8]^ In this study, 2 weeks after the interlaminar ESI, the NRS pain scores decreased by 2.1 points. The reason for this relatively small reduction in pain scores might have been associated with the differences in the medications used (triamcinolone, betamethasone, and dexamethasone), and in the patients’ demographic characteristics. Moreover, the patients had similar pain scores at baseline, independent of their SS severity; these patients presented at a pain clinic for pain management rather than for other types of intervention, which may account for these similarities.

This study has some limitations. First, this was a single-center observational study. However, to reduce selection bias, we used restrictive eligibility criteria and included patients who were similar in age, lifestyle, and baseline pain scores. Second, this study assessed short-term outcomes. Previous studies have suggested that the effects of an ESI are short-lived and may disappear within 3 weeks to 6 months after the injection.^[3,15,18]^ Third, the outcomes considered in this study, such as NRS pain scores and walking distance, were subjective. However, in this study, we focused on improving symptoms (pain reduction and restoration of walking function) in patients with lumbar spinal central stenosis. Prospective randomized controlled trials are required to investigate the effect of the ESI in patients with lumbar foraminal stenosis.

## 5 Conclusion

In conclusion, lumbar interlaminar ESI may reduce pain scores and increase walking distance in patients with moderate lumbar spinal central canal stenosis. In the present study, patients with moderate SS presented with outcomes that were better than those of patients with severe stenosis.

## Acknowledgments

This study has received no specific funding. We would like to thank Editage (www.editage.co.kr) for English language editing.

## Author contributions

**Conceptualization:** Byeongmun Hwang, Minsoo Kim.

**Data curation:** Minsoo Kim, Soyeon Cho.

**Formal analysis:** Hee-Jeong Son, Jin Huh, Seong-Sik Kang.

**Investigation:** Daehun Goh, Soyeon Cho, Yeonji Noh.

**Supervision:** Byeongmun Hwang.

**Writing – original draft:** Byeongmun Hwang.

**Writing – review & editing:** Byeongmun Hwang.
